# The underestimated burden of monogenic kidney disease in adults waitlisted for kidney transplantation

**DOI:** 10.1038/s41436-021-01127-8

**Published:** 2021-03-12

**Authors:** Eva Schrezenmeier, Elisa Kremerskothen, Fabian Halleck, Oliver Staeck, Lutz Liefeldt, Mira Choi, Markus Schüler, Ulrike Weber, Nadine Bachmann, Maik Grohmann, Timo Wagner, Klemens Budde, Carsten Bergmann

**Affiliations:** 1 Charité-Universitätsmedizin Berlin, Department of Nephrology and Medical Intensive Care, Berlin, Germany; 2 Berlin Institute of Health (BIH), Berlin, Germany; 3 KfH kidney center Berlin Moabit, Berlin, Germany; 4 Medizinische Genetik Mainz, Limbach Genetics GmbH, Mainz, Germany; 5 Department of Medicine, Nephrology, University Hospital Freiburg, Freiburg, Germany

## Abstract

**Purpose:**

Chronic kidney disease (CKD) is a major health-care burden. Increasing evidence suggests that a considerable proportion of patients are affected by a monogenic kidney disorder.

**Methods:**

In this study, the kidney transplantation waiting list at the Charité was screened for patients with undetermined cause of CKD. By next-generation sequencing (NGS) we targeted all 600 genes described and associated with kidney disease or allied disorders.

**Results:**

In total, 635 patients were investigated. Of these, 245 individuals had a known cause of CKD (38.5%) of which 119 had a proven genetic disease (e.g., ADPKD, Alport). The other 340 patients (53.5%) were classified as undetermined diagnosis, of whom 87 had kidney failure (KF) onset <40 years. To this latter group genetic testing was offered as well as to those patients (*n* = 29) with focal segmental glomerulosclerosis (FSGS) and all individuals (*n* = 21) suspicious for thrombotic microangiopathy (TMA) in kidney biopsy. We detected diagnostic variants in 26 of 126 patients (20.6%) of which 14 of 126 (11.1%) were pathogenic or likely pathogenic. In another 12 of 126 (9.5%) patients, variants of unknown significance (VUS) were detected.

**Conclusion:**

Our study demonstrates the diagnostic value of comprehensive genetic testing among patients with undetermined CKD.

## INTRODUCTION

In a high proportion of patients with kidney failure (KF) an underlying cause has never been determined. In large registry data, the proportion of patients with an undetermined diagnosis has been reported to be between 20% and 25%^1,2^. In smaller cohorts, the percentage of these patients has been reported to be up to 40%^3^. Usually, patients who do not have a positive family history for kidney disease or a systemic inflammatory disease present late in the progression of chronic kidney disease (CKD) because of its clinically silent character. While a kidney biopsy is still regarded to be part of the diagnostic spectrum for many kidney diseases, there is increasing critical data when and whether to make use of this invasive procedure, especially when no meaningful result is expected. Furthermore, many patients are still often classified as hypertensive glomerulopathy when hypertension and proteinuric kidney disease co-occur. However, hypertension may just be the consequence and not the primary cause of CKD. Beyond patients with autosomal dominant polycystic kidney disease (ADPKD), many individuals suffering from monogenic forms of CKD are misclassified and remain finally undiagnosed. This might be especially true for those patients with unspecific clinical and/or histologic findings. In the past, genetic testing was time-consuming, expensive, and usually only regarded to be promising when a specific clinical phenotype (often with extrarenal features) was present. However, with the introduction and advance of next-generation sequencing (NGS) new possibilities arose and genetic testing became much more cost-effective and accessible^4^. Moreover, turnaround times and detection rates are now much more attractive for the clinical setting than some years ago. Recent data have shown that the number of monogenic disease-causing variants among patients with CKD is about 20–30%^5,6^.

In particular, renal transplantation benefits from knowledge of the underlying kidney disease, not only for the selection and potential exclusion of any potential living donor, but also for the management of these patients before and after transplantation. Despite recent progress, genetic testing of waitlisted patients with undetermined kidney disease is still not routinely performed. This single center study aimed to identify patients with prior undetermined genetic primary kidney disease among patients with KF awaiting kidney transplantation by a comprehensive NGS panel approach.

## MATERIALS AND METHODS

### Study design

The study was conducted as a cross-sectional study of the Eurotransplant kidney waiting list at Campus Charité Mitte Berlin. The Charité Berlin ethics committee approved the study (EA2/020/18). All patients gave their written informed consent for genetic testing. Data were collected at the beginning of October 2016 when 635 patients were listed for kidney transplantation. Patients under 18 years of age or who died during the period of data collection were excluded. All patients had either kidney failure with replacement therapy (KFRT) or CKD G5 without kidney replacement therapy and are summarized as patients with KF^7^.

**Fig. 1:**
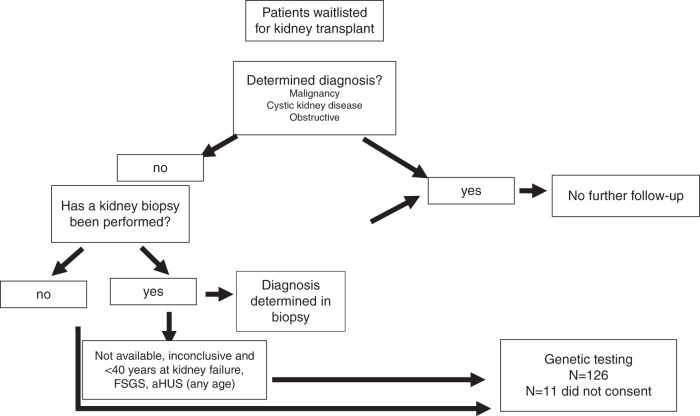
Screening algorithm for patients with kidney failure of unknown origin of the Eurotransplant kidney waiting list at Campus Charité Mitte Berlin. aHUS atypical hemolytic uremic syndrome, FSGS focal segmental glomerulosclerosis.

All patient data were extracted from the patients’ medical files, the hospital records, and transplant database TBase. All patients were screened following the algorithm shown in Fig. 1. Considering the medical history and clinical evaluation, the cohort was divided into cases of determined and undetermined KF. ADPKD was assumed in all patients with a typical radiological presentation of ADPKD. No further genetic testing or a positive family history was required. The other reported genetic kidney diseases have been specified by previously performed genetic analysis, unambiguous syndromic appearance (two patients with prune belly syndrome), hemoglobin electrophoresis (one patient with sickle cell disease) or biochemically, in an individual with cystinosis.

IgA nephropathy, hypertensive nephropathy, diabetic nephropathy, interstitial, amyloidosis, glomerulonephritides, antiglomerular basement membrane glomerulonephritis, systemic lupus erythematosus (SLE), and ANCA-associated vasculitis (AAV) were considered confirmed when seen in a native kidney biopsy. The assignment to obstructive, malignancy, and pyelonephritis was based on evaluation of medical records.

All patients with ambiguous diagnosis due to lack of biopsy or nonconclusive biopsy results were further screened for clinical signs of atypical hemolytic uremic syndrome (aHUS) (thrombocytopenia and hemolysis, KF onset associated with pregnancy or delivery, thrombotic microangiopathy [TMA] [also in allograft biopsies]).

All patients with KF onset <40 years of age and undetermined KF were eligible for genetic testing. Further, all patients with focal segmental glomerulosclerosis (FSGS) as a biopsy result or the suspicion of aHUS due to TMA in a biopsy or other clinical signs were eligible for genetic testing irrespective of age.

### Next-generation sequencing

NGS technologies and comprehensive bioinformatic analyses utilized in this project are described in detail elsewhere^8,9^. In brief, we utilized a customized sequence capture library (e.g., by Twist Bioscience^©^) with curated target regions—currently comprising more than 600 genes described and associated with kidney disease or allied disorders—as well as corresponding flanking intronic sequence according to the manufacturer’s recommendations. The panel design is constantly updated by surveillance of current literature as well as enriched by targets in noncoding regions for described variants listed in well-accepted databases like HGMD or ClinVar. Moreover, the design is optimized in low-performance regions as well as in critical regions like in *PKD1* as described in Eisenberger et al.^9^. DNA samples were pooled and sequenced in a multiplexing procedure. DNAs were enriched using a sequence capture approach, and sequenced using Illumina sequencing-by-synthesis technology with an average coverage of more than 300× for a targeted panel setup. Raw data were processed according to bioinformatics best practice procedures. Mapping and coverage statistics were generated from the mapping output files using standard bioinformatics tools (e.g., Picard). High and reproducible coverage achieved by our sequencing approach enabled copy-number variation (CNV) analysis.

Performance of the wet lab and bioinformatic processes are validated and controlled according to national and international guidelines^10,11^ reaching high sensitivity for single-nucleotide variants (SNVs), indels, and CNVs using well-established reference samples as well as a large cohort of positive controls, especially for CNVs. For interpretation of identified variants, we have developed our own published bioinformatic algorithms using a stepwise filtering process conducted by a very experienced team of scientists and supported by various bioinformatics decision tools. Sequence variants of interest were verified by Sanger sequencing if NGS results failed internal validation guidelines. If other family members were available, segregation of sequence variants with the disease was further assessed.

## RESULTS

The cohort is composed of 635 adult patients (394 males, 241 females) with a median age of 58 years (range 22–88 years). Of these, 526 patients were treated with hemodialysis, 81 patients were treated with peritoneal dialysis, whereas 28 patients were pre-emptively listed for kidney transplantation. The median age at first renal replacement therapy was 49 years (range 18–82 years). A kidney biopsy was available in 297 patients (45.5%). A determined cause of KF was documented in 245/635 (38.5%) patients. In total, 119/635 (18.7%) patients had a genetic disease-causing KF with 104/635 (16.4%) cases of ADPKD, 8/635 (1.3%) cases of Alport syndrome, and 7 cases of rare genetic kidney or syndromic diseases. In our study, the cases with a documented genetic cause of KF were not further investigated by NGS. Of the 126 patients that had a documented nongenetic cause of KF a detailed disease distribution is listed in Supplementary table 1. The majority of patients (340/635 [53.5%]) were classified as having an undetermined diagnosis. Among the patients with undetermined diagnosis, a kidney biopsy has not been performed in 197 patients. In 143 patients a biopsy has been performed, but the result has been inconclusive or the biopsy result was not available since performed decades ago. The median age at first dialysis in the group of patients with determined KF was 50 years (range 18–74 years). The median age of the group of patients with undetermined KF was 49 years (range 20–82 years).

**Fig. 2:**
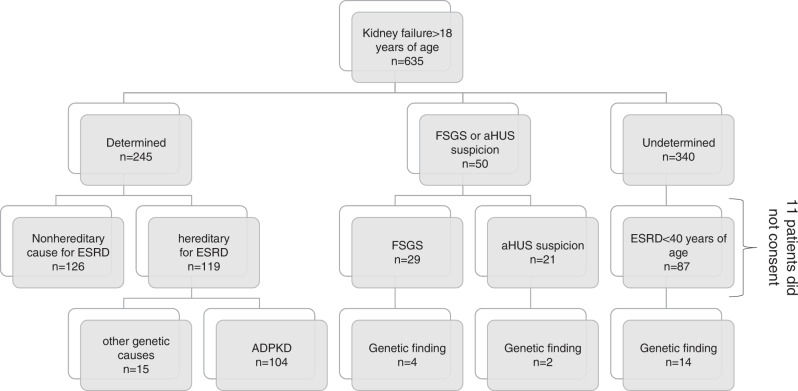
Distribution of genetic and nongenetic causes of kidney failure. ADPKD autosomal dominant polycystic kidney disease, aHUS atypical hemolytic uremic syndrome, ESRD end stage renal disease, FSGS focal segmental glomerulosclerosis.

Of the 390 patients with undetermined diagnosis, 87 were <40 of age at KF onset. In addition, 50 patients suspicious for aHUS or FSGS were eligible for genetic testing. Thus, in total 137 patients met the stringent inclusion criteria for genetic testing of our study. Overall, genetic testing was performed in 126 patients, 11 patients did not give their consent to genetic testing. Among genotyped individuals, 14/126 (11.1%) patients carried pathogenic or likely pathogenic variants. In another 12/126 (9.5%) patients, variants of unknown significance (VUS) were detected (Fig. 2, Table 1). Of the patients with a performed biopsy and an inconclusive result, 4.2% (6 patients) had a genetic diagnosis after completion of NGS testing.

**Table 1:** Identified putatively pathogenic variants.

Patient ID	Sex	Age at first dialysis	Gene	Variant	Zygosity	ACMG/AMP^26^	Causality
1	F	20	*CD46*	c.286 + 2T>G p.(Lys49_Arg96del)	Hom	P	Causative
2	F	28	*COL4A3*	c.2083G>A p.(Gly695Arg)	Het	P	Causative
3	F	25	*COL4A3*	c.3580del p.(Arg1194Glyfs*27) c.4803del p.(Gly1602Alafs*13)	Het Het	P P	Causative
4	M	32	*COL4A3* *COL4A4*	Exon 3 deletion Exon 4 deletion	Het Het	P P	Causative
5	F	56	*COL4A4*	c.4129C>T p.(Arg1377*)	Het	P	Causative
6	F	41	*COL4A5*	c.3508G>A p.(Gly1170Ser)	Het	P	Causative
7	M	22	*COL4A5*	c.3482G>A p.(Gly1161Glu)	Hem	P	Causative
8	M	42	*INF2*	c.652C>T p.(Arg218Trp)	Het	P	Causative
9	F	44	*INF2*	c.653G>A p.(Arg218Gln)	Het	P	Causative
10	M	21	*TTC21B*	c.626C>T p.(Pro209Leu)	Hom	P	Causative
11	M	18	*TTC21B*	c.626C>T p.(Pro209Leu)	Hom	P	Causative
12	M	33	*COL4A4*	c.481G>C p.(Gly161Arg)	Het	LP	Likely causative
13	M	33	*COL4A5*	c.1708G>A p.(Gly570Arg)	Hem	LP	Likely causative
14	M	25	*NPHP4*	c.1124_1125insCC p.(Ser376Leufs*31) c.3766C>T p.(Gln1256*)	Het Het	LP LP	Likely causative
15	M	34	*CD2AP*	c.682C>T p.(Arg228Trp)	Het	VUS	Possibly causative
16	M	45	*CFI*	c.950G>A p.Arg317Gln	Het	VUS	Possibly causative
17	F	20	*COL4A5*	c.4396C>T p.(Arg1466Cys)	Hem	VUS	Possibly causative
18	F	35	*GLA*	c.352C>T p.(Arg118Cys)	Het	VUS	Possibly causative
19	M	28	*INF2*	c.*1 + 1G>C p.?	Het	VUS	Possibly causative
20	F	21	*INF2*	c.2440G>A p.(Asp814Asn)	Het	VUS	Possibly causative
21	M	21	*LAMB2*	c.2809C>T p.(Arg937Trp)	Hom	VUS	Possibly causative
22	M	18	*LMX1B*	c.1130G>A p.(Arg377His)	Het	VUS	Possibly causative
23	M	49	*MYH9*	c.1730T>C p.(Val577Ala)	Het	VUS	Possibly causative
24	M	24	*PAX2*	c.272C>T p.(Ala91Val)	Het	VUS	Possibly causative
25	F	39	*TRPC6*	c.26C>A p.(Pro9His)	Het	VUS	Possibly causative
26	M	29	*TRPC6*	c.1747A>G p.(Arg583Gly)	Het	VUS	Possibly causative

*ACMG/AMP* American College of Medical Genetics and Genomics/Association for Molecular Pathology, *Hem* hemizygous, *Het* heterozygous, *Hom* homozygous, *LP* likely pathogenic, *P* pathogenic, *VUS* variant of unknown significance.

Among the 14 patients with (likely) pathogenic variants, eight affected individuals harbored defects in genes encoding the collagen □345(IV) trimer. Two variants were located in *COL4A3*, two in *COL4A4*, and one patient carried pathogenic variants in each of the two adjacent *COL4* genes on chromosome 2q36 (patient 4). Another three patients (2 males, 1 female) carried X-linked variants in *COL4A5*. The full clinical picture of Alport syndrome was not present in any of those patients and only two individuals (one with a *COL4A3* and one with a *COL4A5* variant) displayed hearing loss as the most common extrarenal manifestation of *COL4*-related disease. Disease onset was between 25 and 56 years in patients with variants in *COL4A3/4* and between 22 and 41 years in patients with *COL4A5* variants. Three of these pathogenic variants were identified when analyzing patients with FSGS as a biopsy result (2× *COL4A4*, *COL4A5*). Two patients had a positive family history with an affected father in a patient with a *COL4A3* variant and an affected mother and uncle in a patient with a *COL4A5* variant.

Two patients were identified to carry likely pathogenic *INF2* variants. One has a clear positive family history of renal disease with early disease onset (patient 9), whereas the family history of the other patients was unremarkable (patient 8). In two brothers with early onset KF, we identified a homozygous pathogenic variant in *TTC21B*. Their parents are consanguineous (first cousins). This variant was previously described to cause familial FSGS. While one of the brothers displayed severe hyperopia, his brother does not show any evidence for an extrarenal manifestation^12^.

Four individuals were heterozygous for the hypomorphic *NPHS2* variant c.686G>A p.(Arg229Gln), which has been intensively studied and demonstrated to be only pathogenic in cases that harbor a C-terminal dominant-negative pathogenic variant in *trans*^13^. In none of our patients such a change in *NPHS2* was present. As outlined in Supplementary Table 2, in a number of additional variant-negative patients we detected class IV or V (likely) pathogenic variants in the heterozygous state, however, all of those individuals lacked a second convincing change in the respective gene in *trans*.

Of the in total 635 patients, 21 (3.3%) patients were clinically suspicious for aHUS. A detailed description of the patients with aHUS are listed in Supplementary Table 3. In one patient suspicious for aHUS a pathogenic variant was detected and one individual was detected with a VUS. Patient 1 presented with hemolysis and thrombocytopenia and harbored a homozygous pathogenic *CD46* change in the alternative complement system. The result of the kidney biopsy was unfortunately not available. The patient was diagnosed with thrombotic thrombocytopenic purpura (TTP) prior to the genetic diagnosis, although ADAMTS13 activity was 38% and no inhibitory antibodies were found. Overall, all parameters are in accordance as reduced ADAMTS13 activity has been described in patients with aHUS^14^ especially in familial cases^15^. Our patient did not show evidence for a positive family history. While parental samples were not available for segregation, it can be assumed that each parent carries the *CD46* variant in the heterozygous state. However, incomplete penetrance is well known for variants in the complement cascade and explained by a multihit pathomechanism. The other patient (patient 16) was shown to carry a VUS in *CFI*. The biopsy also showed evidence for FSGS combined with signs of TMA. There were no clinical reports describing hemolysis or thrombocytopenia in this individual.

Patient 18 was identified with a VUS in *GLA* known to cause Fabry disease. Since concentric cardiac hypertrophy was previously shown by echocardiography in this patient, clinical workup was initiated after obtaining the genetic result. By this, no further clinical sign of Fabry disease was evident. Measurement of ɑ-galactosidase level yielded a slightly reduced value (11.1 µmol/l/h) while Lyso-Gb3 has been in normal range with 0.8 ng/ml. Overall, clinical findings do not favor a diagnosis of Fabry disease in this patient.

Overall, our genotyping efforts changed the composition of the kidney waitlist and decreased the number of patients with undetermined kidney disease from 390 to 376 cases. The number of clear monogenic causes on our waitlist increased from 119 to 133 corresponding to 20.9% of our waitlist. An additional 12 patients (1.8%) carry class III variants of currently unclear significance that may well have contributed to KF. Segregation studies were aimed but parents were deceased or only one parent was available.

## DISCUSSION

In this study, we could confirm the diagnostic value of genetic testing among patients waitlisted for kidney transplantation. The proportion of patients with undetermined kidney disease has been high with 53.7% in our cohort. This is far above reported rates of about 20% in registry data^1,2,16^. A rigorous workup as well as a strict classification process and the exclusion of clinically possible but not proven diagnoses such as diabetic and hypertensive nephropathy contribute to the high number of undetermined cases in our cohort.

Patients with FSGS are known to show a comparably high rate of monogenic pathogenic variants as previously depicted by Yao et al., demonstrating a diagnostic rate of 11% with a gene panel approach. Others have reported even higher rates (21.4%) in adult cohorts with steroid resistant nephrotic syndrome, among them many patients with FSGS^17^. Results of these studies are in line with our findings, where most of the patients had variants known to cause FSGS-like lesions. Of the overall eight patients with *COL4*-related disease only two of these individuals showed any extrarenal manifestation, but none of the patients displayed the full clinical picture described for Alport syndrome. The high proportion of *COL4*-related disease is in accordance with current literature findings that describe variants in *COL4A3–5* genes as the second most frequent genetic kidney disease^18,19^.

Among patients with TMA (encompassing all primary and secondary diagnoses), the rate of patients with a classical diagnosis of primary aHUS carrying a pathogenic variant in one of the complement genes is comparably low. In a retrospective French cohort of 564 patients with TMA it equaled 1.9%. Whether genetic variants also contribute to TMAs classified as secondary cannot be unequivocally answered from these data^20^. In our cohort, one patient with a clinical suspicion of aHUS was identified with a pathogenic variant in one of the genes that if altered lead to uncontrolled overactivation of the alternative complement pathway. Many of our other cases might be rather considered as secondary aHUS since an association with exogenous triggers such as transplantation, pregnancy, or *Cytomegalovirus* (CMV) infection has been described in these patients respectively. The patient harboring a VUS in the complement system showed FSGS lesions associated with aHUS on renal biopsy. There are several reports in the literature that proteinuric kidney diseases can act as a trigger for aHUS caused by endothelial dysfunction, but also that aHUS itself can reciprocally aggravate proteinuria^21–23^.

This study has some limitations. First, we reported a single center approach and focused on patients with disease manifestations early in life. This cutoff excludes all those patients with a disease onset of genetic kidney disease only later in life and thereby likely milder, so-called hypomorphic variants. There is increasing evidence that pathogenic variants can also be identified in elderly patients. Notably, 1/3 of the cohort described by Groopman et al. in their seminal study in the *New England Journal of Medicine* were >65 years of age. Second, we utilized a targeted panel approach. As intensively discussed^4^, there are pros and cons of not only a gene panel, but also an exome-wide approach. The former has the advantage that it will not yield incidental findings in genes unrelated to the primary indication for testing, but also the disadvantage that updating of enrichment-based panels with any newly discovered relevant genes requires a cumbersome redesign and validation of the assay^6^. Thus, virtual phenotype-related gene panels are primarily exome-based and filtered only afterward by respective bioinformatic tools. This exome-based approach is attractive to diagnostic laboratories as it is less cumbersome and allows dynamic gene content update with minimal efforts for design and validation. However, major disadvantages of using exome-based virtual gene panels is that “off the shelf” exome data tend to have less uniform sequence coverage than customized well-balanced, targeted gene panels. Thus, there might be some gaps in important genes that need to be filled by additional sequencing efforts. For a long time, CNVs have been underestimated in their relevance and represent important causes of inherited renal diseases. Sophisticated bioinformatics is necessary to detect CNVs from NGS data, but usually this is more valid from customized gene panel approaches than from exome sequencing. In addition, several important renal disease-causing genes such as the major genes for ADPKD (*PKD1*) and aHUS (*CFH* and its related *CFHR* genes) are located in the most genomically complex regions that require a specific targeted design in order to not miss any disease-causing variant in these genes^9,24^. Thus, we put major efforts in our NGS methodology to find a compromise and established a customized gene panel that targets all 600 genes described and associated with kidney disease or allied disorders—as well as corresponding flanking intronic sequences. The panel design is constantly updated by surveillance of current literature as well as enriched by targets in noncoding regions for described variants listed in well-accepted databases like HGMD or ClinVar. Moreover, our design is optimized in low-performance regions as well as in critical regions like in *PKD1* and *CFH* as described previously and is validated for CNV detection as well.

In our cohort, even more cases could have been classified as nephropathy of unknown origin with a possible genetic origin^25^. For example, eight patients diagnosed with interstitial nephritis in our study could be included in a high-risk population for monogenic kidney diseases, since Groopman et al. reported a genetic disorder in 4.5% of patients with tubulointerstitial disease^6^. Similar studies to ours in the literature often also included patients for genotyping with a clinical diagnosis of ADPKD, Alport syndrome, and/or other rare genetic kidney or syndromic diseases. As is well known, the variant detection rate among those patient cohorts is very high, but much more heterogeneous than still often assumed. Despite these comparably strict inclusion criteria, our approach yielded a genetic diagnosis in more than 20% of cases. As discussed, the detection rate would be significantly higher if patients with determined KF among waitlisted individuals had been incorporated in our study design. Further, the costs are a major obstacle to genetic testing in many parts of the world and were another reason for the age limit of 40 years in our cohort. While the costs for genetic testing are still much higher than for most other diagnostic procedures, fortunately, the cost of NGS is constantly falling and hopefully genetic testing will be soon affordable to more patients and health-care systems. Overall, there is increasing evidence that early use of genetic testing is cost-effective because secondary costs, e.g., related to late diagnosis of disease, are much higher than costs caused by genetic diagnostic testing.

Our study demonstrates the value of customized comprehensive renal gene panel testing among waitlisted patients. The obtained results had direct clinical consequences for some of the affected individuals, e.g., in case of complement variants where respective complement blockers should be used after transplantation or in cases where a specialized workup is indicated. Moreover, extrarenal features of some genetic disorders often remain undetected for many years, but clearly benefit from early detection. For other individuals indirect consequences arise from the obtained results such as concise family counseling. Overall, unequivocal characterization of the underlying disease is of utmost importance with regard to prognosis, clinical management, and the risk of recurrence after transplantation. In contrast to patients with immune-mediated diseases, the prognosis in individuals with most genetic kidney disorders is good with a lack of recurrence. Moreover, therapeutic approaches with steroids and immune suppressive drugs are useless or even contraindicated in patients who harbor a pathogenic variant in an onco- or tumor suppressor gene. Likewise, kidney donation by a family member carrying a dominant pathogenic variant is contraindicated and needs to be avoided. In contrast, heterozygous donors for typical autosomal recessive disorders (in which heterozygotes with a pathogenic variant lack any disease manifestation) can be accepted, which underlines the importance and benefit of a close interdisciplinary collaboration between nephrologist and geneticist.

## Supplementary information

Supplement

## Data Availability

The data that support the findings of this study are available on request from the corresponding author.
