# Correction to: The underestimated burden of monogenic kidney disease in adults waitlisted for kidney transplantation

**DOI:** 10.1038/s41436-021-01283-x

**Published:** 2021-07-23

**Authors:** Eva Schrezenmeier, Elisa Kremerskothen, Fabian Halleck, Oliver Staeck, Lutz Liefeldt, Mira Choi, Markus Schüler, Ulrike Weber, Nadine Bachmann, Maik Grohmann, Timo Wagner, Klemens Budde, Carsten Bergmann

**Affiliations:** 1 Charité-Universitätsmedizin Berlin, Department of Nephrology and Medical Intensive Care, Berlin, Germany; 2 Berlin Institute of Health (BIH), Berlin, Germany; 3 KfH kidney center Berlin Moabit, Berlin, Germany; 4 Medizinische Genetik Mainz, Limbach Genetics GmbH, Mainz, Germany; 5 Department of Medicine, Nephrology, University Hospital Freiburg, Freiburg, Germany

Correction to: *Genetics in Medicine* **23**:2021; 10.1038/s41436-021-01127-8; published online 12 March 2021

Unfortunately, the Funding was given incorrectly.

The following funding should be added: E.S. received a grant from Alexion Pharmaceuticals.

The original article has been corrected.

