# Supplementary material for: The underestimated burden of monogenic kidney disease in adults waitlisted for kidney transplantation
Source: Genet Med. 2021 Mar 12;23(7):1219–24. doi: 10.1038/s41436-021-01127-8 (PMC8257480; doi:10.1038/s41436-021-01127-8)
Supplement: Supplementary file 1 — Supplement [file 41436_2021_1127_MOESM1_ESM.docx]

Supplementary Table 1: Determined diagnosis of non-genetic causes for KF.

Supplementary Table 2: Pathogenic or likely pathogenic variants in the heterozygous state in recessive disease genes and no further convincing variant *in trans*.

Supplementary Table 3: Clinical features of patients with aHUS suspicion.

| Supplementary Table 1: Determined diagnosis of non-genetic causes for kidney failure |  |
| --- | --- |
| IgA nephropathy | 30 |
| Obstructive nephropathy | 16 |
| Hypertensive nephropathy | 13 |
| Diabetic nephropathy | 11 |
| Interstitial nephropathy | 8 |
| Glomerulonephritis other | 8 |
| Malignancy | 8 |
| Anti-GBM-nephritis | 5 |
| Systemic Lupus Erythematosus | 5 |
| Other | 12 |

| Supplementary Table 2: Pathogenic or likely pathogenic variants in the heterozygous state in recessive disease genes and no further convincing variant *in trans* | | | | | | |
| --- | --- | --- | --- | --- | --- | --- |
| Patient-ID | **Sex** | **Age at first dialysis** | **Gene** | **Variant** | **Zygosity** | **ACMG** |
| 27 | m | 47 | *LAMB2* | c.1978_1979del p.(Lys660Glyfs*2) | het | P |
| 28 | f | 28 | *NUP93* | c.1162C>T p.(Arg388Trp) | het | LP |
| 29 | m | 34 | *PLCE1* | c.1223G>T p.(Arg408Ile) | het | LP |
| 30 | f | 36 | *SMARCAL1* | c.2114C>T p.(Thr705Ile) | het | P |

| Supplementary Table 3: Clinical features of patients with aHUS suspicion | | | | | |
| --- | --- | --- | --- | --- | --- |
| Age at first dialysis | **sex** | **Result of renal biopsy** | **TMA in allograft biopsy** | **Associated factors** | **Variant** |
| 54 | m | TMA, arteriosclerosis, tubular atrophy, glomerulosclerosis |  |  |  |
| 31 | f | IgA nephropathy, TMA |  | aHUS occurred after abortion |  |
| 20 | m | n.a. | TMA in renal allograft | aHUS associated with CMV infection |  |
| 35 | f | malignant nephrosclerosis, HUS |  |  |  |
| 47 | m | TMA, diabetic glomerulopathy |  |  |  |
| 40 | m | Intimasclerosis, TMA |  |  |  |
| 41 | m | TMA, malignant nephrosclerosis |  | possibly medication triggered by mefloquine |  |
| 30 | f | FSGS, TMA |  | Antiphospholipid antibodies positive |  |
| 36 | f | IgA nephropathy, TMA |  |  |  |
| 45 | m | FSGS, TMA |  |  | CFI |
| 57 | m | TMA, arteriosclerosis, glomerulosclerosis |  |  |  |
| 44 | m | n.a. | TMA in renal allograft |  |  |
| 20 | f | TMA |  | preeclampsia in pregnancy |  |
| 41 | m | TMA, arteriosclerosis, tubular atrophy, glomerulosclerosis |  |  |  |
| 26 | f | TMA |  |  |  |
| 39 | f | FSGS, TMA |  |  |  |
| 31 | m | n.a. | TMA in renal allograft | aHUS associated with CMV infection |  |
| 26 | m | TMA |  |  |  |
| 36 | m | n.a. | TMA in renal allograft |  |  |
| 53 | m | TMA |  |  |  |
| 20 | f | n.a. |  | ADAMTS13 activity 38% | CD46 |
| aHUS: atypical hemolytic uremic syndrome, TMA: thrombotic microangiopathy, FSGS: focal-segmental glomerulosclerosis, CMV: cytomegalie virus, ADAMTS: a disintegrin and metalloproteinase with thrombospondin 1 like elements, n.a.: not available | | | | | |
